# Keeping nurses in nursing: a qualitative study of German nurses’ perceptions of push and pull factors to leave or stay in the profession

**DOI:** 10.1186/s12912-022-00822-4

**Published:** 2022-02-23

**Authors:** Catharina Roth, Michel Wensing, Amanda Breckner, Cornelia Mahler, Katja Krug, Sarah Berger

**Affiliations:** 1 Department of General Practice and Health Services Research, Heidelberg University Hospital, Marsilius Arcades, West Tower, Im Neuenheimer Feld 130, 69120 Heidelberg, Germany; 2 Department of Nursing Science, University Hospital Tuebingen, Hoppe-Seyler-Str. 9, 72076 Tuebingen, Germany; 3 Centre for Postgraduate Nursing Studies, University of Otago-Christchurch Campus, 2 Riccarton Ave, Christchurch, 9140 New Zealand

**Keywords:** Health workforce, Nursing, Workforce management, Germany, Qualitative research

## Abstract

**Background:**

The increasing nursing shortages worldwide has focused attention on the need to find more effective ways to recruit and retain nurses. The aim of this study was to gain understanding of factors that keep German nurses in nursing and explore their perceptions of factors that contribute to nurses leaving or staying in the profession.

**Methods:**

An explorative qualitative study was undertaken at four different hospitals (two university hospitals and two public hospitals) in Baden-Wuerttemberg, a state in South Germany. Semi-structured face-to-face or telephone interviews were conducted with 21 state-qualified nurses who had graduated from a German nursing program. Each interview was pseudonymized and transcribed. Transcripts were coded according to Qualitative Content Analysis with data structured into themes and subthemes. The study was reported according to the Consolidated Criteria for Reporting Qualitative Studies (COREQ) checklist for qualitative research.

**Results:**

Two themes emerged from the analysis and each theme had several subthemes: a) PUSH FACTORS i.e. factors that may push nurses to consider leaving the profession included limited career prospects, generational barriers, poor public image of nursing, and workplace pressures; b) PULL FACTORS i.e. factors that nurses wished for and could keep them in the profession included professional pride, improved remuneration, recognition of nursing, professionalisation, and improving the image of nursing as a profession.

**Conclusion:**

The decision to leave or stay in nursing is influenced by a complex range of dynamic push and pull factors. Nurse Managers responsible for stabilizing the workforce and maintaining their health system will continue to have to navigate challenges until working conditions, appropriate wages and career development opportunities are addressed. A key to tackling nursing shortages may be focusing on pull factors and nurse managers listening in particular to the perspectives of junior nurses directly involved in patient care, as giving them opportunity to further develop professionally, reinforcing a strong and supportive workplace relationships, paying an appropriate salary, and improving the public image of nursing profession.

**Registration number:**

The study has been prospectively registered (27 June 2019) at the German Clinical Trial Register (DRKS00017465).

## Background

Health systems globally are facing a crisis of workforce shortages [1, 2]. According to the World Health Organisation (WHO), the International Council of Nurses (ICN), and Nursing Now (global campaign run in collaboration with the International Council of Nurses and the World Health Organization) there is a global nursing shortage of 5.9 million nurses, with the greatest need of qualified nurses in South East Asia and Africa [3, 4]. In the European Region, around 7.3 million nurses and midwives are currently employed, however, this number is not adequate to meet current and future needs [5]. Although Germany has the highest number (13.9 nurses) of nurses per 1000 inhabitants in the European Union (OECD (2021)), it has been struggling also to address the increasing need for qualified nurses [6]. A study by the Prognos AG [7], for example, predicted a nursing shortage of 520,000 full-time nurses in Germany in 2030.

The shortage of nurses can be viewed in terms of real nursing shortage and pseudo-shortage. The latter refers to a sufficient availability of qualified nurses, but low willingness to work under present conditions, which results in the decision to leave practice or the profession [2]. Nursing shortages differ by specialty, country, healthcare sector, healthcare service, and organisation [2]. Primary contributing factors are the increased demand for nurses and decreased supply worldwide. Factors linked to increased demand include an aging population, globalization and a growing private sector, and increased social mobility [2]. Unsatisfactory working conditions (e.g., high workloads, inadequate support staff, work-related stress, and workforce burnout) [8], insufficient remuneration, lack of participation in decision making, lack of leadership support, and changes in health human resources approaches are factors associated with a decreased supply of qualified nurses [2, 9, 10]. The demographic changes, particularly in Europe [11, 12], the high prevalence of chronic diseases [13], the fact that more and more qualified nurses are close to retirement [12, 14], and a decreasing number of student nurses puts an increased pressure on the healthcare system [2, 12]. In addition, the shortage of nurses is exacerbated by an aging nursing workforce [12, 15]. Studies have shown that nursing workforce shortages increase the risk of adverse events, nurse-sensitive outcomes e.g. pneumonia or falls, and mortality [12, 16–18], which all have direct impact on the quality of patients care and patient safety [16].

Internationally, several strategies to address the nursing workforce shortage have been undertaken. This includes measures to improve working conditions, expand the recruitment base, and target qualified nurses who have left the nursing profession to return, recruitment of internationally trained nurses, and improved remuneration [19, 20]. However, previous studies have shown that financial incentives are only one of many drivers of nursing shortage [21]; the underlying causes of the global nursing shortages are complex [22]. Strategies that have been applied to date internationally to tackle the increasing nursing workforce shortage seem to be inadequate. Many countries are not able maintain supply of qualified nurses including Germany [6]. The shortage of qualified nurses has been becoming increasingly acute due to increased demand especially in developed countries with aging populations and an aging workforce. The demand for healthcare will continue to increase, while the supply of qualified healthcare staff to meet those needs is going to be limited [6]. The urgent need for newly qualified nurses and also the retention of experienced nurses [23] requires immediate attention. Measures to address these issues need to be implemented in a concerted manner. Improved understanding of factors that keep nurses in nursing may be a key to improving recruitment and retention. In the light of an aging nursing workforce and fewer student nurses, further evidence is needed to identify what attracts young Germans to enter the nursing profession, and what keeps them nursing.

To date, research on turnover and nursing workforce shortages in Germany have mainly focused on assessing factors that influence intention to leave workplace. Few studies have examined factors that keep nurses in nursing, attract young Germans to entering nursing profession, and what the wish for in order to stay. Gaining a better understanding of perceptions of factors that contribute to nurses leaving or staying in the profession in Germany by qualified nurses directly involved in patient care is key to development of successful strategies for future recruitment and retention.

### Study aim

The aim of this study was to advance understanding of factors that keep German nurses in nursing and explore their perceptions of factors that contribute to nurses leaving or staying in the profession.

## Methods

### Study design

The research presented in this manuscript is part of a larger research project (the *Nurse Migration Project)*, conducted by the Department of General Practice and Health Services Research of the University Hospital Heidelberg, Germany. The main aim of the research project *Nurse Migration* was to explore the integration process of internationally trained nurses into the German nursing workforce from the perspective of German trained nurses (GTN) and internationally trained nurses (ITN). Another aim was to gain more in-depth knowledge regarding the experiences of GTN and ITN in the workplace.

The present study reports the findings from a qualitative interview study with GTN with data collected by semi-structured face to face or telephone interviews.

### Study setting

Four German hospitals were invited to participate in this study:

- Centre 1 (University Hospital) has 57 specialized clinical departments with 1.600 beds in total. These services provide high-quality inpatient treatment with best practice medical and nursing standards. Currently, 2601 nurses are employed at Centre 1.
- Centre 2 is (with 310 beds in total) one of the largest lung care clinics in Europe and currently employs more than 200 nurses.
- The Centre 3 works closely with the Centre 1 and has six different departments with around 200 beds in total and employs more than 200 nurses.
- The Centre 4 is a public acute hospital (with 234 beds) which provides basic and standard medical care and currently employs around 280 nurses.

### Participants

Nurses were eligible to participate in this interview study if they were at least 18 years old and were employed in one of the involved hospitals. Other healthcare professionals (e.g., physicians, physiotherapies, nurse aides), as well as student nurses and nurses who did not consent to participate were excluded from the present interview study. No minimum employment working hours was set. All nurses gave their written informed consent to participate in the study prior to the start of the interview.

### Sampling and recruitment process

The nursing leaders of the four hospitals were initially informed about the purpose of the study and the recruitment process by a member of the research team via email and by phone. All hospitals decided to participate in the research project. Different sampling and recruitment methods were applied at the four remaining hospitals.

At Centre 1, a key contact person was appointed by the Director of Nursing management. The key contact person was responsible for informing the ward managers about the nature and the purpose of this study. At Centre 2, all ward nurse managers were informed during a meeting with a member of the research team, that was organized by the nursing management. At Centre 1 and Centre 2 the ward nurse managers were responsible for the distribution of the study material. Each nurse received an envelope with information resources including an invitation letter to take part in the study, an information leaflet, an informed consent form, and a reply envelope for return in the internal mail system. The information leaflet included contact details of the research team. Nurses who decided to take part were requested to contact the research team directly.

At Centre 3 and Centre 4 nursing management informed ward nurse managers about the study. Ward managers then informed their nursing teams and decided together with their teams who was eligible and was willing to participate. They then informed the research team about the interested nurses. Those nurses also received information resources including an invitation letter to take part in the study, an information leaflet, an informed consent form, and a reply envelope via the internal mail system. Together with the interested nurses, the ward nurse manager and a member of the research team arrange interview appointments.

Four weeks and six weeks after the first distribution of the study material reminders were sent to the nursing management and ward managers of each hospital to increase the respondent’s rate. Ward managers were asked to make the survey public at regular team meetings. In addition, nurses who took part in an interview were asked to invite their peers in all four hospitals.

### Data collection

Semi-structured individual face-to-face or telephone interviews were conducted between September 2019 to August 2020 by a female health services researcher with a background in nursing and public health (CR). The interview guide was initially developed by CR (female health services researcher with a background in nursing and public health) and discussed in a qualitative research colloquium at the Department of General Practice and Health Services Research, Heidelberg University Hospital. Adjustments were made according to recommendations by the participants of the colloquium. The interview questions were open-ended and based on an extensive literature search. Interview questions addressed experiences and perceptions regarding workplace experiences. The data collection process was interrupted by the SARS-CoV-2 pandemic in 2020 and was therefore prolonged. Face-to-face interviews were conducted in a separate and quiet room on the ward. All interviews were digitally recorded, pseudonymized and transcribed verbatim. Each transcript was reviewed whilst listening to the digital recording to ensure accuracy. Data gathering was finalized when saturation was reached. Information such as sociodemographic data, work experience, and place of work were collected in addition to the interviews. No additional field notes were taken during or after the interview. Interviews were not repeated. Transcripts were not returned to participants for verification or feedback. Only the participants and the researcher were present during the interview.

### Data analysis

Data was analysed according to Qualitative Content Analysis [24] to structure collected data into themes and subthemes using an inductive approach. In a first step, two female researchers (CR and AB) familiarised themselves with the whole data set. They coded the first three interviews independently. The results were discussed, and a coding system was developed by consensus. The transcripts were then coded line-by-line by CR (health services researcher with background in nursing) and AB (health services researcher). The coded transcripts were compared against the coding system in further discussions. Disagreements regarding codes were resolved in discussions with CR, AB and SB (health services researcher with background in nursing). All transcripts were analysed using the same method by the two coders. Moreover, the final coding system including themes and subthemes and illustrative quotes were discussed between CR, AB and SB in order to ensure consensus as part of the quality management process for qualitative data analysis. Interview data was analysed using MAXQDA, version 2020.1.0 [25], a computer-assisted qualitative data management software.

Quotations presented in this paper were translated into English and slightly adapted to maintain meaning by CR (fluent in German and English) and checked for accuracy by SB (a native English speaker fluent in German).

### Ethical considerations

The study was approved by the Medical Ethics Committee of the Medical Faculty of Heidelberg University (S-367/2019). In addition, the staff council of each hospitals approved the study. Written informed consent was provided prior commitment to interviews by participants. Research conducted in this study was performed in accordance with the Declaration of Helsinki [26]. The study was reported according to the Consolidated Criteria for Reporting Qualitative Studies (COREQ) checklist for qualitative research [27].

### Management of data quality

Rigorous procedures were implemented to enhance the credibility of findings: (a) using more than one data coder during data analysis, (b) peer debriefing (qualitative research colloquium), (c) consensus discussion between the two coders and if necessary, a senior researcher, and (d) member checking/ respondent validation with a senior researcher. In addition, an audit trail of the research process was developed to document the research process [28].

## Results

Twenty-one interviews with German trained nurses were conducted. The majority of nurses were employed at Centre 1. The mean age was 40.4 years (SD 11.4), with the youngest participating nurse being 22 years old and the oldest being 60 years old. More than three-quarters of the total sample identified as female (81.0%). They had work experience of 19.5 years on average. Participants were employed in a variety of different departments. The majority worked on inpatient wards with a small number of nurses from on an intensive care unit (Table 1). Mean interview duration was 17.25 min (range 8.14–23.41).

**Table 1:** Sociodemographic characteristics of the nurses who participated in this study (*n* = 21)

	German trained nurses ***n*** = 21 (100%)
**Study Centre**	**n (%)**
Centre 1	14 (66.7)
Centre 2	2 (9.5)
Centre 3	4 (19.0)
Centre 4	1 (4.8)
**Age in years**	**mean (SD)**
	40.4 (11.4) Min 22 Max 60
**Gender**	**n (%)**
Female	17 (81.0)
Male	4 (19.0)
**Work experience in years**	**mean (SD)**
	19.5 (12.8) Min 0 Max 44
**Role**	**n (%)**
State-Qualified-Nurse	14 (66.7)
Charge Nurse	7 (33.3)
**Department**	**n (%)**
Department of Cardiology and Pneumology	3 (14.3)
Pneumology Department	2 (9.5)
Surgical Department	6 (28.6)
Department of Internal Medicine	5 (23.8)
Gastro-Enterology Department	2 (9.5)
Other	3 (14.3)
**Ward**	**n (%)**
Intensive Care Unit	5 (23.8)
Intermediate Care	1 (4.8)
Inpatient Ward	15 (71.4)

Two main themes emerged from qualitative content analyses in relation to nursing workforce shortage: a) Push Factors and b) Pull Factors (Table 2). Each theme included subthemes. Exemplar quotations were used to illustrate meaning and themes emerging from the considerable data set that was generated from the study. Exemplar quotes were anonymised to protect the identity of participants.

**Table 2:** Overview of themes and sub-themes

Themes	Definition	Subthemes
**Push Factors**	This theme included factors that may push nurses to consider leaving the profession as demotivators from the perspective of nurses trained in Germany	• Limited Career Prospects • Generational Barriers • Poor Public Image • Workplace Pressure
**Pull Factors**	This theme included factors that could keep nurses in the profession or factors that nurses wished for in order to stay as motivators from the perspective of nurses trained in Germany.	• Professional Pride • Improved remuneration • Recognition of Nursing • Professionalisation • Marketing

### Theme 1: push factors

This theme included factors that may push nurses to consider leaving the profession as demotivators from the perspective of nurses trained in Germany.

#### Limited career prospects

Some nurses indicated that a lack of opportunities for professional advancement may be demotivating and thus contribute to nursing shortages in Germany. Participants emphasized the limited opportunities for professional advancement.

*I think it would be more lucrative. Because then people would also come in and say: "Well, I studied” […] and maybe I can develop a bit more diversely […] to study nursing science or nursing management would perhaps be a bit better, I hope, if you do it for a year longer. (Nurse_26, Age 29)*

However, it was also acknowledged that just the higher qualification alone would not always be enough and that there was no easy answer.

*It's just difficult to get promoted, although I also have to say: Well, in the past there was no university degree for nursing. As I said, I see it critically. I don't know... I think at the management level it is good, it's okay if someone has a university degree [...] and then maybe also a degree in business administration, but whether or not a ward manager necessarily has to have an advanced training course or a university degree, I honestly don't know. (Nurse_24, Age 48)*

#### Generational barriers

Some participants described perceived differences between the younger and the older generation of nurses, which involved divergent values and motivations. Some younger nurses highlighted that they wished to have the chance to influence their workplace and have a voice, but they felt their group was too small to initiate change. They had the impression that older nurses have a different mindset and that it was sometimes challenging to find common ground between the two generations, which could be demotivating at work.

*And that is not in the minds of the nurses, that is mainly a generational problem. It works better with the younger ones [younger nurses], it's easy to get them on board, but my generation or my level of education is not yet in a position to perceive it at all. Very few people are, that's my experience. And I think that... they are... Many of them [older nurses] just want to do their work in peace, […] they want to do their work in the best possible way in the area where they feel comfortable. I think that's the difficulty, to get the generations together a bit. (Nurse_22, Age 40)*

#### Poor public image

Participants reported that perceptions in German society had of the nursing profession such as the stereotypical picture of nurses given in the media contributed to a poor public image. They highlighted that many people did not realise the range of professional skills nursing required to provide patient care and how demanding the nursing job really was and that this had a demotivating effect.

*[...] because the nursing profession has been dragged through the mud and degraded, […] we support and remove natural needs from the human being. That's nothing bad and nothing disgusting in my eyes, but one should just, because that was put in the foreground, yes, just the nurse was degraded to the rear cleaner and that's what society has now and it's just yes: "Give food, a bit of washing and yes and therefore they still get too little money for that." (Nurse_31, Age 36)*

*Personally speaking, I think it would be much more beneficial for the nursing profession if we showed a certain amount of transparency. It is... I notice that in my patients, there are many who have no idea about what we actually do here. All the hospital series like "Grey's Anatomy" and I don't know what else, we have already been asked whether we really have relationships with our physicians. Yes, so you have to listen to things like that. Of course, it is completely misrepresented, even if you watch private channels like SWR or ARD and all the others, that this is not the job or the day of a nurse. There are very few nurses who stand up and say: "No, we do much more than just supporting patients with personal hygiene. We do much more than just prophylaxis." (Nurse_26, Age 29)*

Participants described the nursing profession as a highly specialised field that has a wide spectrum of responsibilities and wide range of competencies. Participants stressed that they felt a need to show for society to better understand what nurses do and what they are capable of in order to increase the attractiveness of the profession.

*[…] because many people think: "The doctors are always there", but especially at an intensive care unit, the physicians are usually not at patient’s bedside, [..] the nursing staff actually do everything, they are responsible for the ventilation of patient and things like that […] You also have a lot of responsibility for it, so I think that's more appealing somehow if people would know that […]. (Nurse_18, Age 30)*

#### Workplace pressure

The workplace pressures linked to inadequate staffing were not going to improve if even gaining entry to nursing training was perceived as a barrier for some. Participants indicated that some nursing schools required a secondary school certificate to be able to enrol in nursing school, which could be a barrier for some otherwise suitable candidates for the nursing profession. Participants described the negative impact on their motivation to work due to inflexible rostering choices. They suggested a more family-friendly roster.

*[…] more flexible working hours, more possibilities, everything is still too rigid. (Nurse_22, Age 40)*

Participants described workplace pressure that had a demotivating effect included inadequate staffing levels, constant time pressure during patient care, physical stress, inflexible rostering choices, the impact of political decisions, and managing the physical and emotional work.

*And this time pressure that we have here because we learn to give everything for our patients during nursing training, this and that, hygiene also needs a certain amount of time, yes, and we also need time for conversations, but it is not possible, because otherwise you simply won't get through and won't get any further, yes, that is very difficult. […] We work with people and very often we forget that, because there is simply pressure from above and that is just a pity, because that is not what we have learned in this profession, […] and at some point, after the time I say: "I can't do any more." Yes, I cannot spend 24 hours here doing what I do. I just have to do the bare minimum and that's not why I'm a nurse. (Nurse_25, Age 30)*

### Theme 2: pull factors

This theme included factors that could keep nurses in the profession or factors that nurses wished for in order to stay as motivator from the perspective of nurses trained in Germany.

#### Professional pride

Participants emphasized the importance of a sense of pride for their profession. These nurses described their feelings of pride, and that nursing work was meaningful and contributed to society.

*To be proud of what we have learned and what we are doing as nursing profession, […]. (Nurse_24, Age 48)*

*And that's where I say, "Ok we are nurses, we just have to learn to be happy with it." [...] Of course more money is great; of course, more recognition is great. But […] it just helps me to say: "Hey, I'm proud of my profession it is not just a job, it's a vocation". (Nurse_26, Age 29)*

#### Improved remuneration

Participating nurses discussed factors related to remuneration or salary as a pull factor for staying in the workforce. They highlighted that higher payment could raise the status of the profession. In addition, improved remuneration would show nurses that they were valued and that their work was appreciated.

*In order to address the shortage of skilled workers in nursing, I see above all an attractive salary that expresses a certain appreciation for nursing staff and that should definitely become apparent, which will perhaps be a factor for many to return to the nursing profession. (Nurse_10, Age 29)*

However, some participants indicated that a better salary alone was not enough to increase the attractiveness of the nursing profession.

*I think that more money alone doesn't make it [nursing] more attractive, at least not in the long term. (Nurse_29, Age 42)*

*Yes, and I mean payment is always quite nice and good, but I think the work has to be fun. Of course, you have to be able to make a living from it, but it also has to be fun […]. (Nurse_24, Age 48)*

#### Recognition of nursing

Participants stressed that the recognition of nursing started with how they sew themselves but also how other healthcare professionals and society saw them. They indicated that in order to be recognised as a profession they had to show the world what it meant to be a nurse and what the world would be like without nurses. Factors participants thought that positively impacted on the image of nursing were a strong social standing, being proud of being a nurse, and a professional association that worked towards maintaining high status of the nursing profession.

*I think that it is a shame and I have a lot of criticism for the society in general, but especially now with nursing, it starts with us, so if someone young were to ask me, a young woman who is perhaps thinking about becoming a nurse, I would advise her to (Nurse_8, Age 56)*

Participant were in favour of collaborating with other healthcare professionals to find best practice solutions. They highlighted the desire to improve collaboration and increase recognition of nursing by other professions.

*I think it would help if we worked more in an interdisciplinary team, I personally suffer from the hierarchical structures and sometimes I think I've been working as nurse for so long and I'm always here, but I'm just not allowed to have a say in ward decisions […] I find really bad, and that's also something that probably won't change in the time I'm there, but for the future I hope that there will be more equality [...]. (Nurse_8, Age 56)*

Another factor mentioned in relation to g the image or recognition of nursing was how nurses talk about their profession themselves. Some participants were concerned that other nursing colleagues had sometimes discouraged young people to choose nursing as profession and wished that their colleagues acted differently.

*It starts with us, so if someone young were to ask me, a young woman who is perhaps thinking of becoming a nurse, I would advise her to do so, well, I would, for example, I've really had good experiences all my life, I've learned a lot for myself, I've managed to raise three children at the same time and I also find that it's always manageable with shift work and with sometimes more and sometimes less work. […] And besides, I think that there are a lot of people working there who are very open-minded and social, good colleagues and I would advise, but I also find that there are a lot of people who say 'Oh, don't do that, don't do that' because of the shift work, so I don't think it's that bad. (Nurse_8, Age 56)*

#### Professionalisation

The advancement of the profession with transition to university qualification was an important pull factor for sustaining the workforce. The majority of participants perceived the opportunity to get a university degree in nursing as important, nevertheless indicating that it would be important to generate jobs including an adequate salary for nurses with a university degree, not only for academics but also for those in clinical practice.

*I also think it is important to build up a mix of qualifications, because I think the professionalisation of nursing is relatively important. In my opinion, this does not have to happen extensively, because perhaps it is not important for everyone, but the possibility of getting a university degree in nursing should exist, and it should then also result in appropriate jobs with appropriate remuneration - that's it. (Nurse_10, Age 29)*

*[…] That's something, you have to get the two fields together, then I think professionalisation has a better chance. If you could get it closer to practice. Most people are moving away from practice, as far as that's concerned, and.... because there are no fields. That's also the thing with... You have to take fields of activity, for example in the clinic, and that's what's still missing. If you don't manage to do that, then I think the nursing profession will develop into the blue-collar sector and the academic sector, and then we'll have a gap that I don't think is really good. (Nurse_22, Age 40)*

Nevertheless, participants stressed that transition to university qualification could not be the only way. They wished that nurses without a university degree also received recognition and appreciation.

*And I see that as a huge problem, so you would have to create something, so professionalisation on the one hand, but on the other hand I would have to create something where I have people who are professionally qualified [without a university degree], where I know they will stay in nursing […]. (Nurse_24, Age 48)*

Some participants indicated that professionalisation could enhance the image of the nursing profession and contribute to workforce retention.

*I think it has to be about a status. With the new Nursing Reform Act, we are perhaps on the right path towards having clearly defined tasks, reserved tasks and no longer being regarded as an auxiliary profession, but as a profession in its own rigth. Of course, it will take a few more years for this to become established, but I think that this is actually a good way forward, as is this university degree, that we are becoming more professional. What I think is important is that we get a skills-mix on the wards, i.e. students with different qualification [pathways e.g. hospital and university training programs] but also support roles such as healthcare assistant, ward secretary, […] Yes, so that a qualified nurse or also a ward nurse manager can then also pursue their activities accordingly, namely the planning, control and organisation of a patient's care needs and control and is involved. (Nurse_29, Age 42)*

#### Marketing

Finally, participants highlighted the need for effective advertising and marketing campaigns targeting young people, improving the image of the different hospitals, job information day at schools, showing presence at job fairs, and increasing positive media presence as another pull factor to attract young people into the workforce.

*Yes, I don't know about job information days at schools or so. I've never actually had nursing anywhere or at these job fairs, yes at Jobs4Future they are, or presentations at the job centre or so, where I used to go nursing was not present, at least I hadn't seen any nurses […], maybe with practical exercises and so, I think you can catch the people. (Nurse_16, Age 22)*

## Discussion

Key findings highlighted that primary push factors for nurses questioning whether to stay in nursing were workplace pressures and poor public image followed by limited career prospects and generational barriers. Moreover, pull factors such as improved remuneration, professionalisation, professional pride, effective marketing and increased social recognition were all factors that could attract young people into nursing and motivate them to stay in nursing as a satisfying and meaningful profession.

Limited career prospects, not having a voice, and not being able to influence ones working environment have been recognised in other studies [29–33]. Flinkman et al. [29], conducted a qualitative case study with young registered nurses in Finland and explored why they intended to leave the nursing profession. Lack of career advancement and the fear of being stuck at one place without being able to further develop were factors that contributed to their decision to change their career. Clendon et al. [30] found that for young nurses in New Zealand career progression (e.g. completing master’s degrees) was important and a factor in retention. Hasselhorn et al. [32] found that nurses in Europe with an intention to leave the profession were usually young, highly qualified and looking for a way to professionally develop. Lynn et al. [33] explored solutions to the nursing shortage from the point of view of nurses working in eight different states in the USA. Career progression and educational opportunities were key motivators to stay in the profession [33]. In addition, studies conducted in the USA suggested that giving nurses a voice to influence their work environment resulted in increased satisfaction and retention [34, 35]. In light of an aging nursing workforce, the loss of highly motivated and qualified (young) nurses due to limited career prospects and lack of new challenges is unacceptable.

Nurses in our study, particularly the younger generation, indicated that the opportunity to obtain a university degree was important to them for realising their professional potential. In addition, limited career prospects were perceived as a push factors for considering leaving the profession. The commitment and wish to gain experience and skills is typical for millennial generation nurses [36]. Shields et al. [37], examined the impact of job satisfaction of British nurses on intention to quit and found that dissatisfaction with promotion and training opportunities are found to have a stronger impact than workload or insufficient remuneration. A study conducted in Ethiopia found that nurses with a bachelor or a master degree scored higher on the motivation score compared to nurses with less education [38]. Watts et al. [39], found that the intention to leave was lower in certified nurses than in non-certified nurses.

Differences between younger generations and the older generations of nurses are also an important consideration. Particularly, the younger generation of nurses participating in our study were enthusiastic and excited about their chosen career. They highlighted their desire to shape their workplace and further develop professionally but felt held back by those from an older generation. These results reflect the findings by Clendon et al. [30] and Dols et al. [40]. Retention strategies that enabled young nurses to be heard and have an opportunity to advance in their career through for example professional development or project work are necessary. However, these strategies will only be effective in the long term if nurses are paid accordingly and have sufficient time and resources needed to do their work [30].

In our study nurses, described stereotypical portrayals of nurses in the media that contributed to poor public image. They felt that the nursing profession was undervalued by other professions and society in general in Germany. These findings are supported by the work of other researchers [29, 41, 42]. Historically, the nursing profession has been a female profession and has been associated with stereotypical attributes such as being the submissive handmaids of dominant physicians with little responsibility [42–44]. Particularly, young nurses in our study did not identify with this picture. They rather saw themselves as highly skilled and ambitious professionals. This is consistent with findings by Flinkman et al. [29] and Takase et al [41]. .Takase et al. [41], conducted a study investigating the impact of the perceived public image of nursing on nurses’ work behaviour in Australia and found that the poor public image can decrease self-esteem and lead to job turnover.

Previous research has already established that workplace demands have a significant impact on the physical and emotional health of nurses and their intention to leave the nursing profession [22]. Findings in our study highlighted that high workplace demands and poor practice environment were reasons for intention to leave, which is consistent with those by Huntington et al. [45], Haywards et al. [46], and Flinkman et al. [29]. In a multi-national study with nurses working in New Zealand, Australia, and the United Kingdom high workload, constant pressure, and staff shortages had a negative impact on the job satisfaction of nurses and increased intention to leave [45]. Flinkman et al. [29], also identified workplace pressure as a major driver for young nurses to leave the nursing profession especially those linked to insufficient practice environment and nurse-patient ration. In an Canadian study [46], factors that influenced experienced nurses’ decision to leave their profession included increased workload due to a higher number of acutely ill and sicker patients.

Not being able to provide a high standard of patient care and the ensuing moral distress was a reason for some nurses in our study to think about leaving the profession. Nurses experiencing emotional and physical exhaustion due to the inability to provide a reasonable level of care over time leads to moral distress that in turn prompts nurses to consider leaving their profession [29, 45–47]. There are growing demands of the nursing profession as patient acuity increases linked to longer lifespans of people with long-term conditions and multimorbidity. Nurses need to be able to fulfil the requirements of modern patient care by applying evidence-based interventions. However, in order for nurses to be willing to stay in nursing, workplace conditions need to be such that they have the ability to provide patient care without experiencing chronic emotional and physical exhaustion.

Given the challenges face by nurses in contemporary clinical practice, which threaten further losses to an already dwindling workforce, a clear understanding of factors that keep nurses in nursing is essential. In our study positive workplace relationships contributed to a positive feeling and increased job satisfaction, which has also been reported elsewhere [45, 46]. Fair and appropriate remuneration is another strong motivating factor. Nurses in a Swedish study [31] indicated, that an unsatisfactory salary was the main reason for leaving the nursing profession. Flinkman et al. [29] found that nurses thought their salary was not adequate, and that a higher salary would improve attractiveness of nursing [29]. However, improved remuneration alone does not compensate if other factors are unsatisfactory. Cox et al. [48], reported that moral distress and poor interpersonal relations were the primary mediating factor for turnover rather than pay. Improved remuneration alone is not a solution to nursing workforce shortages but are part of a bundle of strategies needed to improve the image of nursing and increase nurse satisfaction, so they stay in nursing.

Similar to findings in our study, Hayward et al. [46], described the lack of respect in their working relationships with physicians contributed to nurses decision to leave their jobs. Rosenstein et al [49], .conducted a survey in the USA investigating the effect of nurse-physician relationship on nurse satisfaction and intention to leave. Disruptive behaviour from physicians increased work-related stress and frustration [49]. This behaviour was not only experienced in physicians-nurse relationships but also in nurses-nurse relationships [49]. Our findings also indicated that the way nurses talk with each other particularly about their own profession may have a negative impact on retention or recruitment. Rosenstein et al. [49] showed that the prevalence of disruptive behaviour resulted in nurses resigning [49]. Nurses seek to be recognized and wish for positive workplace relationships not only with their peers but also other healthcare professionals. Improving collaborative interprofessional teamwork may be another key to improved interprofessional relationships and thus enhance the recognition of the nursing profession. Integration of interprofessional education into the education of different healthcare professionals may be a strategy to address this issue.

## Conclusion

The decision to leave or stay in nursing is influenced by a complex range of dynamic push and pull factors. Nurse Managers responsible for stabilizing the workforce and maintaining their health system will continue to have to navigate challenges until working conditions, appropriate wages and career development opportunities are addressed.

### Implications for practice

A global undersupply of nursing and attrition rates that continue to climb means that nurse managers and policymakers need clear understanding of a range of financial, professional, and personal factors that keep nurses in nursing. Particularly, young nurses globally are looking for opportunities to develop professionally. It is important to them to have the opportunity to qualify at a university and gain increased autonomy. Strong and supportive workplace relationships seem to be one of the key factors to keep nurses in nursing not only in Germany but globally. Inadequate remuneration and the poor public image of the nursing profession seem to contribute to the intention to leave nursing profession. In addition, not being able to provide adequate patient care seem to be one of the main factors globally that leads to nurse turnover, particularly in young nurses. In order to address the nursing shortages, further research is needed to explore what young nurses are looking for, what motivates them to choose nursing, and what keeps them nursing worldwide.

### Strengths and limitations

This study explored on factors that may push nurses to consider leaving the profession and pull factors that may keep them in nursing. In contrast to previous studies, which mostly focused on factors that contributed to intention to leave the nursing profession, this study provided a perspective on factors that kept nurses in nursing. Qualitative interviews are an important research tool to gain in-depth knowledge on the research subject and understanding of perspectives of targeted groups and was therefore considered as appropriate research method. Data analysis was guided by adequate methodological strategies aiming to minimize bias and reduce the risk of losing relevant content. Reporting of the qualitative findings was guided by the recommendation of the COREQ checklist [27].

Some limitations must be acknowledged. Although the qualitative design allowed an in-depth exploration of the nurses’ perceptions on push and pull factors, findings of this study cannot be generalized beyond the study population. In addition, as the study was conducted in South Germany in two university hospitals and two smaller public hospitals, specific national and regional factors might have influenced the results. A similar study conducted elsewhere might get different results due to context. Even though data saturation was reached, higher number of nurses from different hospitals may have led to more diverse results. Although perceptions and experiences were relatively consistent within the study sample, these experiences may not be shared by all nurses. In addition, nurses who voluntary participated might have different perceptions and experiences compared nurses that choose not to participate. Social desirability in answers cannot be excluded. Quotes were translated from German into English; it is therefore possible that the meaning in translated quotes subtlely differed from the original meaning in German quotes. The results of the study must be interpreted with caution in terms of generalization and representativeness.

## Data Availability

The dataset that was generated and analysed during the study will not be made publicly available due to German data protection law but may be made available by the corresponding author on reasonable request.

## References

[1] Littlejohn L, Campbell J, Collins-McNeil J (2012). Comparative analysis of nursing shortage. Int J Nurs.

[2] Oulton JA (2006). The global nursing shortage: an overview of issues and actions. Policy Politics Nurs Pract.

[3] Nursing and midwifery - Fact sheets: World Health Organization 2020. Available from: https://www.who.int/news-room/fact-sheets/detail/nursing-and-midwifery. [updated 9 January 2020; cited 2021 19 April]

[4] WHO and partners call for urgent investment in nurses: World Health Organization 2020 [updated 7 April 2020; cited 2022 17 February]. Available from: https://www.who.int/news/item/07-04-2020-who-and-partners-call-for-urgent-investment-in-nurses.

[5] Nursing and Midwifery - Data and statistics: World Health Organization; 2021. Available from: https://www.euro.who.int/en/health-topics/Health-systems/nursing-and-midwifery/data-and-statistics. [cited 2021 19 April]

[6] Reiff E, Gade C, Böhlich S (2020). Handling the shortage of nurses in Germany: opportunities and challenges of recruiting nursing staff from abroad.

[7] Pflegelandschaft 2030 (2021). Studie der Prognos AG im Auftrag der vbw - Vereinigung der Bayerischen Wirtschaft e V. 2012.

[8] Lu H, Barriball KL, Zhang X, While AE (2012). Job satisfaction among hospital nurses revisited: a systematic review. Int J Nurs Stud.

[9] Hayes LJ, O'Brien-Pallas L, Duffield C, Shamian J, Buchan J, Hughes F (2012). Nurse turnover: a literature review - an update. Int J Nurs Stud.

[10] van der Heijden BI, Kümmerling A, van Dam K, van der Schoot E, Estryn-Béhar M, Hasselhorn HM (2010). The impact of social support upon intention to leave among female nurses in Europe: secondary analysis of data from the NEXT survey. Int J Nurs Stud.

[11] Linz K, Stula S (2010). Demographic change in Europe-an overview. Observ Sociopolit Dev Europe.

[12] Duvall J, Andrews D (2010). Using a structured review of the literature to identify key factors associated with the current nursing shortage. J Prof Nurs.

[13] Busse R, Blümel M (2010). Tackling chronic disease in Europe: strategies, interventions and challenges: WHO regional office Europe.

[14] Sherman RO, Chiang-Hanisko L, Koszalinski R (2013). The ageing nursing workforce: a global challenge. Wiley Online Library.

[15] Camerino D, Conway P, van der Heijden B, Estryn-Behar D, Consonni D, Gould D (2006). Low-perceived work ability, ageing and intention to leave nursing: a comparison among 10 European countries. J Adv Nurs.

[16] Aiken LH, Clarke SP, Sloane DM, Sochalski J, Silber JH (2002). Hospital nurse staffing and patient mortality, nurse burnout, and job dissatisfaction. JAMA..

[17] Griffiths P (2009). Staffing levels and patient outcomes. Nurs Manag (Harrow).

[18] O'Brien-Pallas L, Murphy GT, Shamian J, Li X, Hayes LJ (2010). Impact and determinants of nurse turnover: a pan-Canadian study. J Nurs Manag.

[19] Konzertierte Aktion Pflege: Erster Bericht [press release] (2020). Bundesministerium für Familie, Senioren, Frauen und Jugend.

[20] Buchan J (2001). Nurse migration and international Recruitmen. Nurs Inq.

[21] Milisen K, Abraham I, Siebens K, Darras E, de Casterlé BD (2006). Work environment and workforce problems: a cross-sectional questionnaire survey of hospital nurses in Belgium. Int J Nurs Stud.

[22] Aiken LH, Clarke SP, Sloane DM, Sochalski JA, Busse R, Clarke H (2001). Nurses’ reports on hospital care in five countries. Health Aff.

[23] Duffield CM, Roche MA, Blay N, Stasa H (2011). Nursing unit managers, staff retention and the work environment. J Clin Nurs.

[24] Schiener J (2010). Sozialforschung. Methoden und Anwendungen. Ein Überblick für die BA-Studiengänge. Uwe Flick, 2009. Methods Data Anal.

[25] Kuckartz U, Rädiker S. Introduction: analyzing qualitative data with software. In: Analyzing Qualitative Data with MAXQDA. Cham: Springer; 2019. p. 1–11. 10.1007/978-3-030-15671-8_1.

[26] Association WM (2013). World medical Association declaration of Helsinki: ethical principles for medical research involving human subjects. JAMA..

[27] Tong A, Sainsbury P, Craig J (2007). Consolidated criteria for reporting qualitative research (COREQ): a 32-item checklist for interviews and focus groups. Int J Qual Health Care.

[28] Lincoln YS, Guba EG (1985). Naturalistic inquiry.

[29] Flinkman M, Isopahkala-Bouret U, Salanterä S (2013). Young registered Nurses' intention to leave the profession and professional turnover in early career: a qualitative case study. ISRN Nurs.

[30] Clendon J, Walker L (2012). Being young': a qualitative study of younger nurses' experiences in the workplace. Int Nurs Rev.

[31] Fochsen G, Sjögren K, Josephson M, Lagerström M (2005). Factors contributing to the decision to leave nursing care: a study among Swedish nursing personnel. J Nurs Manag.

[32] Hasselhorn H, Tackenberg P, Müller H (2003). Work conditions and intent to leave the profession among nursing staff in Europe.

[33] Lynn M, Redman R (2006). Staff nurses and their solutions to the nursing shortage. West J Nurs Res.

[34] Ulrich B, Lavandero R, Hart K, Woods D, Leggett J, Taylor D (2006). Critical care nurses’ work environments: a baseline status report. Crit Care Nurse.

[35] Lake E, Friese C (2006). Variations in nursing practice environments: relation to staffing and hospital characteristics. Nurs Res.

[36] Broom C (2010). Entice, engage, endure: adapting evidence-based retention strategies to a new generation of nurses. J Healthc Leadership.

[37] Shields MA, Ward M (2001). Improving nurse retention in the National Health Service in England: the impact of job satisfaction on intentions to quit. J Health Econ.

[38] Negussie N (2012). Relationship between rewards and nurses' work motivation in Addis ababa hospitals. Ethiop J Health Sci.

[39] Watts MD (2010). Certification and clinical ladder as the impetus for professional development. Crit Care Nurs Q.

[40] Dols J, Landrum P, Wieck KL (2010). Leading and managing an intergenerational workforce. Creat Nurs.

[41] Takase M, Maude P, Manias E (2006). Impact of the perceived public image of nursing on nurses' work behaviour. J Adv Nurs.

[42] Seago JA, Spetz J, Alvarado A, Keane D, Grumbach K (2006). The nursing shortage: is it really about image?. J Healthc Manag.

[43] Darbyshire P, Gordon S (2005). Exploring Popular Images and Representations of Nurses and Nursing.

[44] Fletcher K (2007). Image: changing how women nurses think about themselves. Liter Rev J Adv Nurs.

[45] Huntington A, Gilmour J, Tuckett A, Neville S, Wilson D, Turner C (2011). Is anybody listening? A qualitative study of nurses' reflections on practice. J Clin Nurs.

[46] Hayward D, Bungay V, Wolff AC, MacDonald V (2016). A qualitative study of experienced nurses' voluntary turnover: learning from their perspectives. J Clin Nurs.

[47] McGillis Hall L, Kiesners D (2005). A narrative approach to understanding the nursing work environment in Canada. Soc Sci Med.

[48] Cox KB (2001). The effects of unit morale and interpersonal relations on conflict in the nursing unit. J Adv Nurs.

[49] Rosenstein A, O'Daniel M (2005). Disruptive behavior & clinical outcomes: perceptions of Nurses & Physicians. Am J Nurs.

